# Dr. Bernard’s Experiments on the Elective Elimination of Certain Substances by the Secretions

**Published:** 1853-05

**Authors:** 


### RECORD OF MEDICAL SCIENCE.

### MATERIA MEDICA AND THERAPEUTICS.

*Dr. Bernard's Experiments on the Elective Elimination of certain Substances by the Secretions.*—Dr. Cl. Bernard has written a very interesting account of certain experiments made by him "on the elective elimination of certain substances by the secretions, and in particular by the salivary secretion." He remarks *in limine* that this secretion has not been examined with the same care as the urine, bile, milk, &c., in regard of the circumstance alluded to; and that it remains to be explained how the saliva chooses some, while it rejects other substances equally soluble in it. He performed two sets of experiments, which were ingeniously contrived and carefully repeated.

In the first series of experiments he injected into the right jugular vein a solution of yellow prussiate of potash, of iodide of potassium, and of grape sugar, and immediately thereafter he detected the second substance in the saliva, but neither of the other two; while in the urine, the prussiate of potash could be detected, but neither the iodide nor the sugar. Twenty-five minutes after the injection, abundance of the prussiate of potash was found in the urine, only a trace of sugar, but still no iodide. The saliva remained as before. The secretions were tested every half-hour, but no change occurred till the end of two hours, from the time after injection, when the iodide of potassium at length appeared in the urine. Neither the prussiate nor the sugar appeared at any time in the saliva, which eliminated only the iodide of potassium; and it is worthy of remark that this salt appeared immediately in the saliva, while it was not detected in the urine for two hours. When, however, the solution of the iodide was stronger, it appeared sooner in the urine, though never within the hour.

In the second series of experiments, M. Bernard injected these three substances in solution, severally into the veins of the same animal at different times, as well as into the veins of different animals, and he always found that they comported themselves in exactly the same manner. Such was the case, likewise, when they were introduced into the stomach. Both grape and cane sugars, like the prussiate of potash, never appeared in the saliva, while they were eliminated more slowly by the urine. This observation seemed to contradict the assertions of some authors, regarding the saliva of diabetic patients, but on actually testing this secretion in 'la Charité,' M. Bernard found that there was no trace of sugar in the saliva, while, however, it could be detected in the expectoration from the bronchi, of such patients as had phthisis combined with diabetes. Neither sugar nor prussiate of potash would seem to pass into the bile, or into the pancreatic juice, in ordinary circumstances, but when sugar was strong in the blood, it was found in the bile, but never in the pancreatic juice.

In regard to the elimination of sugar from the economy, M. Bernard has noted a very curious fact, viz., that though the mammary secretion naturally contains a kind of sugar, it does not allow either cane or grape sugar to pass by it. Sugar of milk is much more difficult of fermentation than the other kinds of sugar, and may thus be distinguished from them.

When the iodide of potassium was injected into the vein of a dog, or introduced into its stomach, it could always be detected in the saliva within forty seconds. It also passed with rapidity into the tears, and into the pancreatic juice, while it passed with much greater slowness into the bile, in which it was often difficult of detection.

M. Bernard also injected lactate of iron into the veins of dogs, and never found it eliminated by the saliva; in which respect it agreed with the sugars and prussiate of potash. When iodide of iron was carefully injected into the vein of a dog, both iodine and iron were found in the saliva. In another dog, a solution of the iodide of potash was introduced into the stomach, by a fistulous opening, and afterwards another solution of lactate of iron; both substances were thereafter detected in the saliva, showing that the iodine gave to the iron the capability of being eliminated by the saliva.

M. Bernard at present merely wishes to call attention to these interesting facts, and does not offer any explanation of them. But the property which certain substances seem to possess, of being eliminated by different secretions, is not their only peculiarity. Their period of sojourn in the economy is also importantly different. Thus, M. Bernard remarks, that the iodide of potassium and other substances, perfectly soluble, and really dissolved in the blood, remain for a certain time within some of the organs of the body; and he finishes his paper with the following account of the experiments made by him, in order to investigate this sojourn of the iodide of potassium in the animal economy.

He introduced into the stomachs of several dogs, which had permanent salivary and biliary fistulæ, a solution of two grammes of iodide of potassium. The same day the urine of these dogs exhibited the reaction of the iodide; next day it could be detected neither in the bile nor in the urine; and on several days following, no trace of it was found in these secretions. It seemed to be completely eliminated from the system; but examination of the saliva showed its presence still. The gastric juice also contained the iodide, both because it contained saliva, and also because it was furnished directly with it from the mucous membrane of the stomach. This persistence of the iodide in the saliva and gastric juice continued for three weeks, and possibly it may have continued longer. Purgatives have a great effect on the sojourn of the iodide in the economy; indeed, so much is this the case, that if purgatives were employed, soon after the introduction of the salt into the stomach, a few days sufficed for its total disappearance from all the secretions.

In concluding, M. Bernard observes, that these experiments show that substances which are soluble, and capable of circulating in the organism without producing mischief, present two sets of phenomena worthy of remark.

"1st. Some substances never pass into certain determinate secretions, *e. g.* the yellow prussiate of potash, cane and grape sugars; others show themselves in all the secretions, only with greater or less rapidity, *e. g.* the iodide of potassium.

"2d. Some of these substances are eliminated completely and rapidly from the economy, *e. g.* the yellow prussiate, sugars, &c.; while others are only *partially* eliminated by the urine, and may remain in the organism, showing themselves in other secretions for a longer or shorter time. The iodide of potassium offers a remarkable example of this prolonged sojourn of soluble substances in the organism,—a sojourn which, in the case of that salt, is prolonged, because the portion not eliminated and re-appearing in the saliva, instead of being expelled from the system is constantly thrown back into the stomach, whence it is taken up by the circulation, and returned to the saliva, and so on.

"The chief conclusion," he continues, "to be drawn from this work is, that one cannot refer to any general law, the manner in which these substances act in the organism. The experiments made on one saline substance, can teach nothing regarding another: no one could have foreseen, for example, that the iodide of potassium, and the yellow prussiate of potash, salts equally soluble, should offer, in respect of their passage into the secretions, and of their elimination from the body, differences very striking. Special researches on each particular substance are necessary, in order to establish physiological history, which ought to be intimately connected with its mode of action as a therapeutic agent."—*Archives Générales de Medecine, January,* 1853.

